# JC Virus Reactivation Should Be Considered in Late Neurotoxicity Associated with Bispecific Antibody Therapy: A Comment. Comment on Bangolo et al. Comprehensive Review of Early and Late Toxicities in CAR T-Cell Therapy and Bispecific Antibody Treatments for Hematologic Malignancies. *Cancers* 2025, *17*, 282

**DOI:** 10.3390/cancers18101506

**Published:** 2026-05-08

**Authors:** Utku Iltar, Unal Atas, Orhan Kemal Yucel, Ozan Salim, Levent Ündar

**Affiliations:** Division of Hematology, Department of Internal Medicine, Faculty of Medicine, Akdeniz University, Antalya 07100, Turkey; vrlunalatas@gmail.com (U.A.); okyucel@hotmail.com (O.K.Y.); ozansalim@gmail.com (O.S.); leventundar@gmail.com (L.Ü.)

We read with great interest the comprehensive review by Bangolo et al. entitled “Comprehensive Review of Early and Late Toxicities in CAR T-Cell Therapy and Bispecific Antibody Treatments for Hematologic Malignancies”, recently published in *Cancers* [1]. The authors should be congratulated for providing an extensive and clinically relevant overview of both early and late toxicities associated with CAR T-cell therapies and bispecific antibodies (BsAbs), particularly highlighting delayed complications that require long-term vigilance.

In the section addressing late complications of bispecific antibody therapy, the authors appropriately note that delayed neurotoxicity may manifest as cognitive impairment, mood disturbances, or chronic neurological symptoms, often outside the classical immune effector cell-associated neurotoxicity syndrome (ICANS) framework. However, we would like to emphasize that opportunistic viral infections—specifically JC virus (JCV) reactivation—should be considered a critical and potentially underrecognized contributor to late-onset neurotoxicity in patients receiving BsAb therapy.

BsAbs, particularly BCMA-directed agents, induce sustained immune modulation characterized by prolonged T-cell engagement, plasma cell depletion, hypogammaglobulinemia, and cumulative immunosuppression. This immunologic environment may predispose patients to a reactivation of latent neurotropic viruses such as JC virus, potentially resulting in progressive multifocal leukoencephalopathy (PML). Importantly, the clinical presentation of PML may initially overlap with or mimic treatment-related neurotoxicity, leading to diagnostic delay and inappropriate management.

In our recently reported case, we described a patient with relapsed/refractory multiple myeloma who developed JC virus-associated PML during elranatamab therapy, confirmed by characteristic magnetic resonance imaging findings and cerebrospinal fluid JC virus polymerase chain reaction positivity [2]. Notably, neurological symptoms emerged after multiple cycles of therapy and outside the typical temporal window of ICANS. Despite prompt discontinuation of elranatamab and supportive interventions, the disease followed an aggressive and ultimately fatal course. This case underscores the devastating consequences of delayed recognition of infectious neurotoxicity in this setting.

Distinguishing PML from immune-mediated neurotoxicity is clinically important because the management approaches differ substantially. Whereas ICANS typically occurs early after treatment initiation, PML may present later and follow a more subacute progressive course. Importantly, late neurotoxicity after BsAb therapy has a broad differential diagnosis, including infection, metabolic disturbances, cerebrovascular events, disease progression, and treatment-related toxicity. Nevertheless, in patients with late, progressive, focal, or otherwise atypical neurological findings, brain magnetic resonance imaging and cerebrospinal fluid analysis including JCV PCR should be considered to help exclude PML. Radiologically, PML often demonstrates multifocal, non-enhancing white matter lesions on T2-FLAIR imaging, in contrast to the often nonspecific or reversible findings described in ICANS.

Emerging reports of JCV reactivation and PML across different BCMA-directed platforms—including teclistamab, elranatamab, other bispecific antibody-based approaches, and ciltacabtagene autoleucel—suggest that profound humoral and cellular immune disruption may represent a shared predisposing context, although this should currently be regarded as a hypothesis rather than a proven class effect [2,3,4,5,6,7].

We believe that future reviews and clinical guidance on late toxicities of BsAbs should explicitly mention JCV reactivation and PML as part of the differential diagnosis for delayed neurotoxicity. Given the limited evidence base, we are not advocating routine JCV screening or surveillance in asymptomatic patients. Rather, we suggest heightened clinical awareness and timely diagnostic evaluation—including neuroimaging and cerebrospinal fluid JCV testing—when clinically indicated.

We again commend Bangolo et al. for their valuable contribution and hope that this perspective further enriches the discussion on late neurological complications associated with bispecific antibody therapies.
